# Screening to Identify Commonly Used Chinese Herbs That Affect *ERBB2* and *ESR1* Gene Expression Using the Human Breast Cancer MCF-7 Cell Line

**DOI:** 10.1155/2014/965486

**Published:** 2014-05-27

**Authors:** Jen-Hwey Chiu, Chun-Ju Chang, Jing-Chong Wu, Hui-Ju Liu, Che-Sheng Wen, Chung-Hua Hsu, Jiun-Liang Chen, Ling-Ming Tseng, Wei-Shone Chen, Yi-Ming Shyr

**Affiliations:** ^1^Institute of Traditional Medicine, School of Medicine, National Yang-Ming University, Taipei 112, Taiwan; ^2^Division of General Surgery, Department of Surgery, Taipei Veterans General Hospital, Taipei 112, Taiwan; ^3^Division of General Surgery, Department of Surgery, Cheng Hsin General Hospital, Taipei 112, Taiwan; ^4^Department of Food Science, National Taiwan Ocean University, Keelung 202, Taiwan; ^5^Center for Traditional Chinese Medicine, Kaohsiung Veterans General Hospital, Kaohsiung 81362, Taiwan; ^6^Department of Orthopedics, Cheng Hsin General Hospital, Taipei 112, Taiwan; ^7^School of Traditional Chinese Medicine, Chang Gung University, Taoyuan 333, Taiwan; ^8^Center for Traditional Chinese Medicine, Chang Gung Memorial Hospital, Taoyuan 333, Taiwan; ^9^School of Medicine, National Yang-Ming University, Taipei 112, Taiwan; ^10^Division of Colorectal Surgery, Department of Surgery, Taipei Veterans General Hospital, Taipei 112, Taiwan; ^11^Experimental Surgery of the Department of Surgery, Taipei Veterans General Hospital, Taipei 112, Taiwan

## Abstract

*Aim*. Our aim the was to screen the commonly used Chinese herbs in order to detect changes in *ERBB2* and *ESR1* gene expression using MCF-7 cells. *Methods*. Using the MCF-7 human breast cancer cell line, cell cytotoxicity and proliferation were evaluated by MTT and trypan blue exclusion assays, respectively. A luciferase reporter assay was established by transient transfecting MCF-7 cells with plasmids containing either the *ERBB2* or the *ESR1* promoter region linked to the luciferase gene. Chinese herbal extracts were used to treat the cells at 24 h after transfection, followed by measurement of their luciferase activity. The screening results were verified by Western blotting to measure HER2 and ER**α** protein expression. *Results*. At concentrations that induced little cytotoxicity, thirteen single herbal extracts and five compound recipes were found to increase either *ERBB2* or *ESR1* luciferase activity. By Western blotting, Si-Wu-Tang, Kuan-Shin-Yin, and Suan-Tsao-Ren-Tang were found to increase either HER2 or ER**α** protein expression. In addition, *Ligusticum chuanxiong* was shown to have a great effect on *ERBB2* gene expression and synergistically with estrogen to stimulate MCF-7 cell growth. *Conclusion*. Our results provide important information that should affect clinical treatment strategies among breast cancer patients who are receiving hormonal or targeted therapies.

## 1. Introduction

Breast cancer is the most common invasive female cancer worldwide as well as in Taiwan [1, 2]. Due to its diverse morphological features, the pathological classification of breast cancers has insufficient prognostic and predictive power. Recently, molecular analysis of breast cancer tissue using immunohistochemistry, measurement of proliferative capacity, and analysis of gene expression profiles has provided a number of feasible treatment strategies that correlate well with clinical outcome [3–5].

There is a consensus that the estrogen and/or progesterone receptor status of breast cancers is an important prognosis factor when assessing the probable response to adjuvant hormonal therapy [6, 7]. Hormonal therapy using tamoxifen over a five-year period has been shown to result in a significant reduction in the annual breast cancer death rate of 34% [8]. In addition, using antibody against the HER2/neu receptor (trastuzumab) or/and the use of its dimerisation inhibitor (pertuzumab) remain important adjuvant target therapies after surgery [9, 10]. Nonetheless, information concerning possible interactions between herbs and drug targets such as the ER receptor and HER2/neu remains to be elucidated.

Accumulating evidence suggests that hormone therapy has a number of adverse effects among breast cancer patients, such as insomnia, hot flushes, and cancer-related fatigue; for example, hot flushes occur in up to 80% of women who are receiving tamoxifen [11–13]. It is generally accepted that the use of complementary and alternative medicines (CAMs) has increased among oncology patients, with the prevalence being as high as 70% to 80% of patients in non-Asian areas [14, 15] and around 36% to 40% in Taiwan. It is worth noting that traditional Chinese medicine (TCM), by definition, cannot be defined as CAMs in oriental countries. In Taiwan, more than one-third of breast cancer patients have used TCM and more than 80% of TCM users chose Chinese herbal products as their means of adjuvant management of breast cancer [16]. Unfortunately, many of these patients who use TCM herbal remedies are not aware of their potential adverse effects, namely, the possibility of herb-drug interactions that might counteract the effects of the hormonal therapy or targeted therapy [17].

For thousands of years, herbal medicines have been an important treatment strategy in traditional Chinese medicine and have been widely used to maintain human health in oriental countries. Epidemiological evidence has shown that many herbal remedies, such as Jia-Wei-Xiao-Yao-San, are commonly used to alleviate severe symptoms in breast cancer patients who are receiving hormonal therapy or targeted therapies [18]. Our previous* in vitro* [17] and* in vivo* studies using MCF-7 breast cancer cells have shown that Si-Wu-Tang (SWT) is able to reverse the antiproliferative effects induced by tamoxifen, including tumor weight, tumor volume, increased ER*α* expression, and N-cadherin expression, when the tamoxifen + SWT-treated group is compared to the tamoxifen-treated group [19]. However, information on whether other commonly used Chinese herbal medicines may affect ER*α* and HER2 expression in breast cancer cells is lacking. Accordingly, the aim of this study was to screen using the human breast cancer MCF-7 cell line a number of commonly used Chinese herbs, which included twenty-two single herbs and six compound recipes, in order to determine whether these herbs/recipes are able to affect * ESR1*  and * ERBB2*  gene expression.

## 2. Materials and Methods

### 2.1. Cell Line and Cell Culture

The MCF-7 (ER+, HER2 low) cell line was obtained from the American Type Culture Collection (ATCC, Manassas, VA, USA). MCF-7 cells were maintained in DMEM supplemented with 10% fetal bovine serum (FBS), 2 mM L-glutamine (Sigma), 1.5 g/L sodium bicarbonate (Sigma), 0.1 mM nonessential amino acid, 1.0 mM sodium pyruvate (Sigma), and penicillin/streptomycin. To prevent the influence of hormones or estrogen-like compounds that may be present in conventional culture medium, the cultured cells were transferred to phenol red-free DMEM containing 5% charcoal-dextran stripped fetal bovine serum (CDFBS), nonessential amino acid, and penicillin/streptomycin 4 days before treatment. Furthermore, the cells were incubated with hormone-free medium and the water extract of the various herbal remedies in the presence of polymyxin B (1 *μ*g/mL) to avoid the possibility of lipopolysaccharide contamination.

### 2.2. Preparation of Extracts from Commonly Used Chinese Herbs (CHEs)

In this study, twenty-two commonly used Chinese single herbs are presented in Table 1 using their scientific names, which are coded from A to V. These are* Astragalus membranaceus *(A),* Atractylodes macrocephala *(B),* Poria cocos* (C),* Glycyrrhiza uralensis* (D),* Agastache rugosa* (E),* Codonopsis pilosula* (F),* Zingiber officinale *(G),* Angelica sinensis* (H),* Ligusticum chuanxiong* (I),* Ziziphus jujuba* (J),* Millettia dielsiana* (K),* Curcuma phaeocaulis* (L),* Folium nelumbinis* (M),* Bupleurum chinense* (N),* Mentha piperita* (O),* Gardenia jasminoides* (P),* Paeonia suffruticosa* (Q),* Taraxacum mongolicum* (R),* Anemarrhena asphodeloides* (S),* Paeonia lactiflora* (T),* Rehmannia glutinosa* (U), and* Ligustrum lucidum* (V). The six compound recipes are as follows, namely, Si-Wu-Tang, Jia-Wei-Xiao-Yao-San (JWXYS), Suan-Zao-Ren-Tang (SZRT) and its reduced formula Suan-Zao-Ren-Tang, K'uan-Hsin-Yin, and VGH-S4 (Table 1). The reduced formula of Suan-Zao-Ren-Tang (r-SZRT) is composed of the same herbs as Suan-Zao-Ren-Tang except for* Ligusticum chuanxiong *which is absent. VGH-S4, which contains* Curcuma phaeocaulis*,* Taraxacum mongolicum*,* Millettia dielsiana,* and* Mentha piperita*, is a compound recipe commonly used in Taipei Veterans General Hospital. JWXYS is composed of* Radix bupleuri*,* Radix Angelicae sinensis*,* Radix Paeoniae alba*,* Rhizoma Atractylodis macrocephalae*,* Poria*,* Rhizoma Zingiberis preparata*,* Cortex Moutan*,* Fructus Gardeniae*,* Herba Menthae,* and* Radix Glycyrrhizae praeparata*. The preparation of extracts from these Chinese medicinal herbs, denoted as CHEs (Chinese herbal extracts) followed standard procedures in order to obtain compositions similar to those used clinically. The herb materials were extracted by a good manufacturing practice (GMP) company (Sun Ten Pharmaceutical Co., Ltd.). The final preparations were stored at −20°C until their use in the experiments. The quality control (QC) of the herbs was monitored by high-performance liquid chromatography (HPLC) [17, 20].

### 2.3. MTT Cytotoxicity Assay and Cell Proliferation Assay

The cytotoxic activity of the various CHEs with respect to MCF-7 cells was determined by the colorimetric assay using 3-(4,5-Dimethylthiazol-2-yl)-2,5-diphenyltetrazolium bromide (MTT). Cells were seeded 24 hr prior to treatment in a 96-well plate at 1 × 10^4^ cells/well. After 24 hr of attachment, cells were treated with various doses (0~10 *μ*g/L) of individual CHEs for 48 and 72 hr. Each CHE was dissolved in phosphate buffered saline (PBS) pH 7.4. And PBS alone was used as the vehicle control. For MTT assay, both the treated and untreated cells were incubated with 100 *μ*L MTT (tetrazolium compounds) for 4 h and lysed with 100 *μ*L DMSO, and the optic density was read using an ELISA reader at a wavelength of 570 nm. In addition, where needed, cell proliferation was determined by the trypan blue exclusion method.

### 2.4. Luciferase Reporter Assays Used to Measure* ERBB2* and* ESR1* Gene Expression Levels

To investigate the effects of commonly used CHEs on* ERBB2* and* ESR1 *gene expression, the luciferase reporter vector pGL2, containing the human HER2 gene (*ERBB2* luciferase: RDB number 2839, RIKEN BioResource Center, Ibaraki, Japan) or the luciferase reporter vector pGL4 containing the human ER**α** gene (*ESR1* luciferase: RDB number 7528) promoters were constructed and transiently transfected into MCF-7 cells. The transfection procedure was performed by following the manufacturer's recommended protocol from the “T-Pro nonliposome transfection reagent II (T-Pro NTRII)” transfection kit (T-Pro Biotechnology, JT97-N002). In brief, 1 × 10^5^ cells/well were seeded for 24 h, which was followed by the following transfection procedure. Plasmid DNA (2**μ**L) or 6**μ**L reagent II was mixed with Opti-MEM individually and then mixed together at RT for 20 min. The mixture was added to individual wells containing cells for 5 hr. The cells were then washed with PBS and medium changed to 1% CDFBS for 19 hr. At 24 hr, luciferase activity was determined. The transfection efficiency was about 90%. In parallel, the Renilla Luciferase Assay System (Promega Corporation, WI, USA) was used for reporter quantification. The results are presented as relative optic density ratios, namely, the ratio of the luciferase activity to the Renilla luciferase activity.

### 2.5. Western Blot Analysis

MCF-7 cells were cultured overnight at a density of 3 × 10^5^ cells/well. The cells were then treated either with a CHE or with vehicle. Next the samples were homogenized using 400 *μ*L lysing buffer containing 150 mmol/L KCl, 10 mM Tris, pH 7.4, 1% Triton X-100, and protease inhibitor cocktail (Complete Mini, Roche, Mannheim, Germany). The protein concentration of each cell homogenates was determined as described previously [21]. Samples consisting of 30–50 *μ*g of protein were separated on 10% SDS-polyacrylamide gels by electrophoresis and thereafter transferred to a PVDF membrane (Millipore, Bedford, MA, USA). The membrane was blocked with 5% bovine serum albumin and probed with specific primary antibodies, namely, anti-ER*α* (Stressgen Biotechnologies Inc., Victoria, BC, Canada), anti-pHER2/anti-tHER2 (IPVH00010, Millipore, Bedford, MA, USA), anti-*α*-tubulin (AbFrontier, Seoul, Korea), and anti-*β*-actin (AbFrontier, Seoul, Korea). The immunoreactive bands were visualized using enhanced chemiluminescence detection reagents (Thermo Scientific, Bremen, Germany) and quantified by Multigauge software analysis (Fuji Photo Film Co., Ltd., Tokyo, Japan).

### 2.6. Statistical Analysis

Data are expressed as the mean ± SEM. Differences between groups at each dose point were identified by one-way ANOVA, followed by Dunnett's* post hoc* test. A *P* value < 0.05 was considered statistically significant compared to vehicle or no treatment group.

## 3. Results

After assessing the cytotoxicity of the commonly used CHEs, which consist of twenty-two single herbs and six compound recipes, we chose a dose range when carrying out further experiments for each CHE where there was ≥80% cell survival (Table 2).

### 3.1. Effects of the CHEs on* ERBB2*-Luciferase Activity

**Figure 1:**
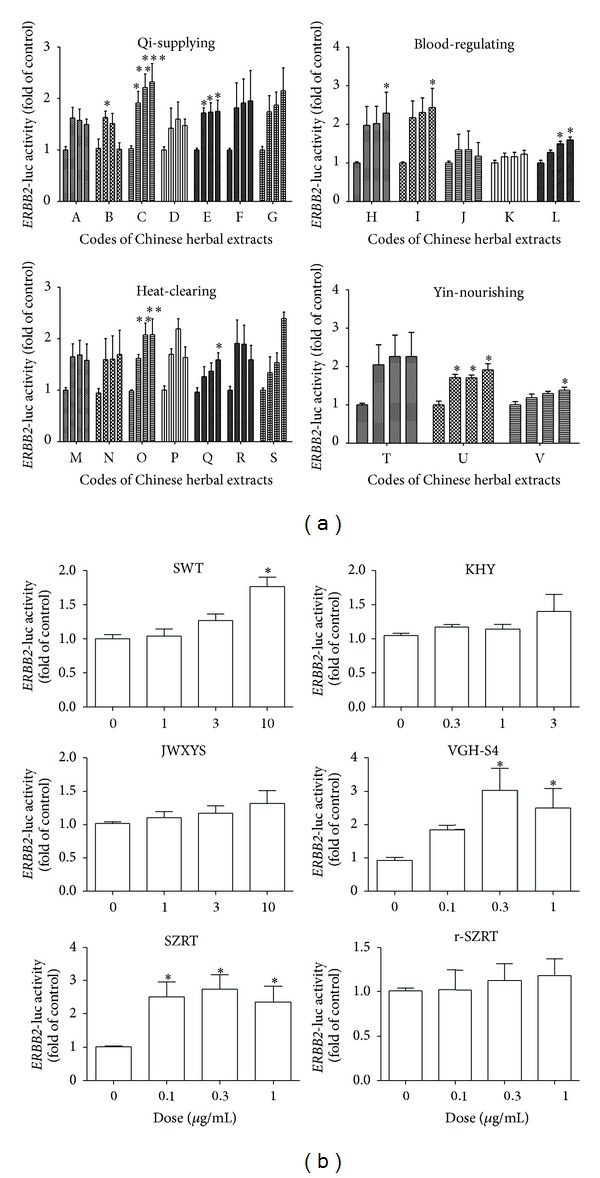
Effects of commonly used Chinese herbal extracts on* ERBB2*-luciferase activity using the MCF-7 cell line. The MCF-7 line (1 × 10^5^ cells/well) was seeded for 24 h, followed by transfection with the* ERBB2*-luciferase plasmid and treatment individually with twenty-two single herbal extracts (a) and six compound recipes (b) as described in Section 2. The codes for the twenty-two herbs (from A to V) are described in Section 2. Data are presented as the relative optic density ratio, namely, the ratio of luciferase activity to Renilla luciferase ratio and were analyzed using four to six independent experiments. An asterisk indicates a *P* value < 0.05 versus the vehicle group by one-way ANOVA.

At levels that caused little or no cytotoxicity, the effect of the CHEs on* ERBB2*-luciferase activity was determined by luciferase reporter assay. The results for the twenty-two herbs (Figure 1(a)) and six compound recipes (Figure 1(b)) are presented in Figure 1.

### 3.2. Effects of the CHEs on* ESR1*-Luciferase Activity

**Figure 2:**
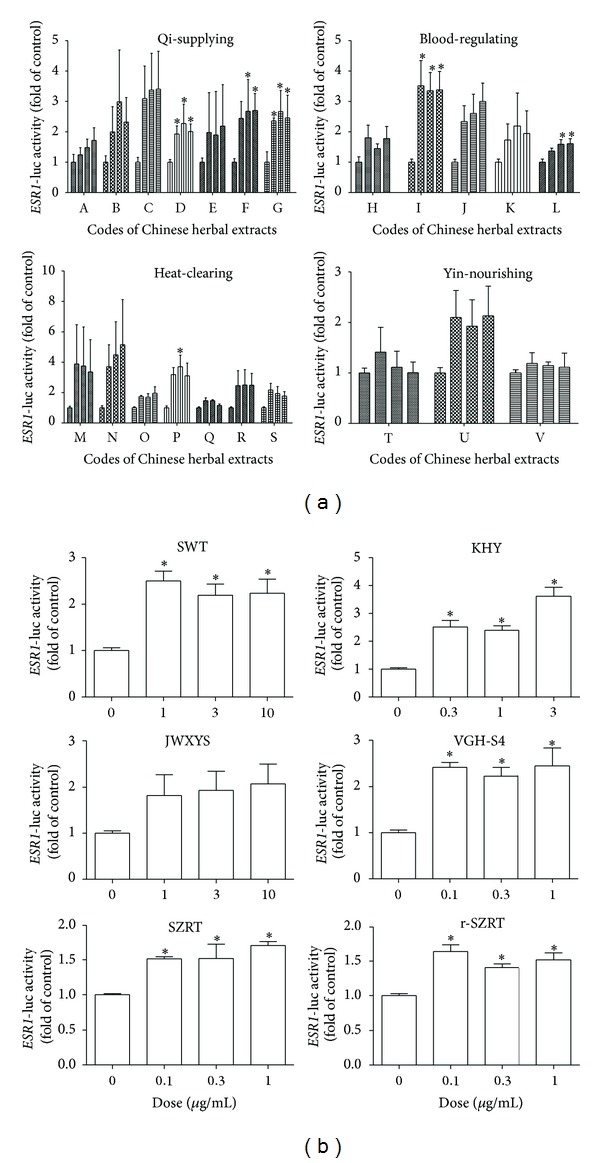
Effects of commonly used Chinese herbal extracts on* ESR1*-luciferase activity using the MCF-7 cell line. The MCF-7 line (1 × 10^5^ cells/well) was seeded for 24 h, followed by transfection with the* ESR1*-luciferase plasmid and treated with twenty-two single herbal extracts (a) and six compound recipes (b) as described in Section 2. The codes of twenty-two herbs (from A to V) are described in Section 2. Data are presented as the relative optic density ratio, namely, the ratio of luciferase activity to Renilla luciferase ratio and were analyzed using four to six independent experiments. An asterisk indicates a *P* value < 0.05 versus the vehicle group by one-way ANOVA.

Similarly, the effect of the CHEs on* ESR1*-luciferase activity was also determined by luciferase reporter assay. The results for the twenty-two herbs (Figure 2(a)) and six compound recipes (Figure 2(b)) are presented in Figure 2.

Since the nature and characteristics of the Chinese herbs studied here are quite different, the CHEs were categorized into four groups, namely, Qi-supplying herbs, blood-regulating herbs, heat-clearing herbs, and Yin-nourishing herbs. Furthermore, the herbs that induced an increase in* ERBB2* and* ESR1 *luciferase activity, after normalization against the vehicle control, were grouped into three quantitative categories, namely ≤1.5, 1.5–2.0, and ≥2.0. The effects of the Qi-supplying and blood-regulating herbs on* ERBB2* and* ESR1* luciferase activity are presented in Table 3, while the effects of the heat-clearing and Yin-nourishing herbs are presented in Table 4. The effects of the compound recipes on* ERBB2* and* ESR1* luciferase activity are presented in Table 5.

### 3.3. Effects of the CHEs on ER*α* and HER2 Protein Expression

**Figure 3:**
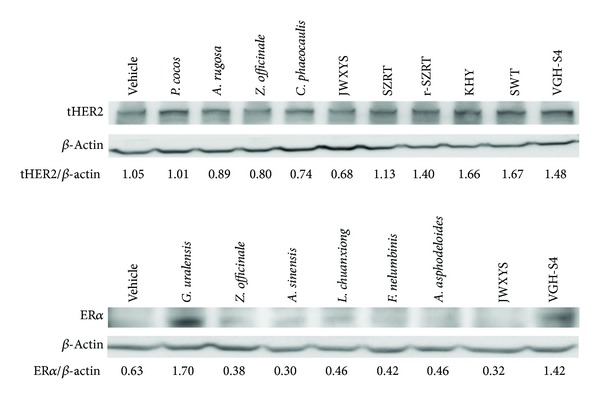
Effects of Chinese herbal extracts on HER2 and ER*α* protein expression using the MCF-7 cell line. The MCF-7 line (1 × 10^6^ cells) was seeded for 24 h and changed to 1% CDFBS medium for 4 days and then treated with each Chinese herbal extract individually for another 24 h. Then the cells were treated with lysing buffer and analyzed by Western blotting using anti-HER2 antibodies (a) and anti-ER*α* antibody (b) as described in Section 2. The OD ratio indicates the relative optic density ratio using *β*-actin as the control.

In order to validate the results obtained from luciferase report assay, the effects of some CHEs on ER*α* and HER2 protein expression were analyzed by Western blotting (Figure 3).

**Figure 4:**
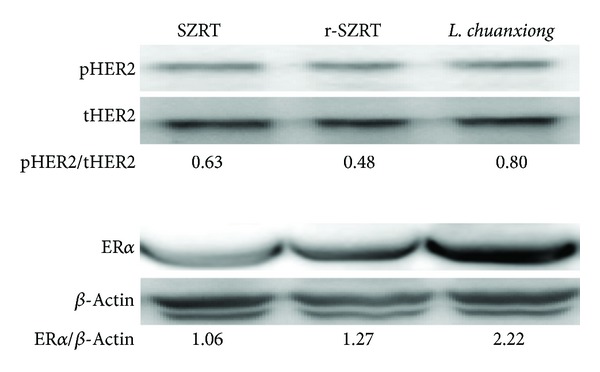
Effects of* Ligusticum chuanxiong* extract on ER*α* and HER2 protein expression using the MCF-7 cell line. The MCF-7 line (1 × 10^6^ cells) was seeded for 24 h and changed to 1% CDFBS medium for 4 days and then treated with Suan-Zao-Ren-Tang (SZRT), the reduced form of Suan-Zao-Ren-Tang (r-SZRT) or* Ligusticum chuanxiong* extracts, for another 24 hr. Then the cells were treated with lysing buffer and analyzed by Western blotting using anti-HER2 antibodies (a) and anti-ER*α* antibody (b) as described in the Methods section. The OD ratio indicates the relative optic density ratio using *β*-actin as the control.

Firstly, the effect of* Ligusticum chuanxiong* extract on ER*α* and HER2 protein expression was explored. Since the reduced form of Suan-Zao-Ren-Tang (r-SZRT) is composed of the same herbs as Suan-Zao-Ren-Tang except for* Ligusticum chuanxiong* which is missing, the effects of SZRT, r-SZRT, and* Ligusticum chuanxiong* on the protein expression levels of the HER2 and ER*α* were compared. The results showed that there was greater induction of pHER2 and ER*α* protein expression by* Ligusticum chuanxiong* alone compared to SZRT and r-SZRT (Figure 4).

**Figure 5:**
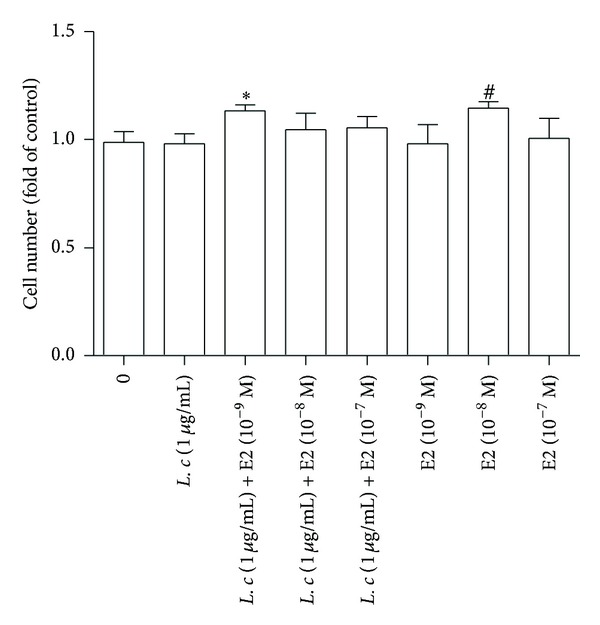
Effects of combined treatment with* Ligusticum chuanxiong* and estrogen (E2) on the cell proliferation of MCF-7 cells. The MCF-7 line (1 × 10^6^ cells) was seeded for 24 h and changed to 1% CDFBS medium for 4 days and then treated with* Ligusticum chuanxiong* extract (1 *μ*g/mL) with or without exogenous estrogen E2 (10^−9^  ~10^−7^ M). This was followed by counting the cell numbers using the trypan blue exclusion method. Data are presented as folds relative to the control and were analyzed using six independent experiments. An asterisk indicates a* P* value < 0.05 versus vehicle group.

Since ER*α* and HER2 play important roles in promoting the growth of breast cancer cells, cell proliferation was also evaluated after treatment of* Ligusticum chuanxiong* extract with or without exogenous estrogen. The results showed that cotreatment of E2 (10^−9^ M) and* Ligusticum chuanxiong* extract (1 *μ*g/mL) stimulated the cell growth of MCF-7 cells, compared to E2 (10^−9^ M) alone or* Ligusticum chuanxiong* extract (1 *μ*g/mL) alone (Figure 5). These findings suggest that* Ligusticum *chuanxiong synergistically increases the effect of estrogen on MCF-7 cell proliferation.

## 4. Discussion

In this study, we have established an* in vitro* screening system that allows us to investigate the effects of commonly used Chinese herbs on* ERBB2* and* ESR1* gene expression. To our knowledge, we are the first to conduct such a study in order to provide important information that will affect clinical practice and the treatment of patients with receptor (+) breast cancer.

There is a consensus that the contamination with lipopolysaccharide (LPS) during herb extraction may influence the interpretation for drug effect data. Thus, pretreatment of the cultured cells with polymyxin B (1 *μ*g/mL) is mandatory in order to avoid the possibility of LPS affecting the results [22]. In addition, charcoal-dextran stripped (CD) fetal bovine serum in phenol red-free medium has been commonly used previously in estrogen-sensitive culture systems in order to avoid the effects of exogenous estrogen [17]. Furthermore, the plasmids containing* ERBB2*-promoter region and the* ESR1*-promotor region with the luciferase gene were validated by direct sequencing and matched against public databases.

Previous studies have demonstrated that some Chinese herbs are able to modulate HER2 expression* in vitro*. For examples, black cohosh,* Shiraia bambusicola*, and honokiol have been shown to suppress the growth of breast cancer cells on the molecular level via an inhibition of HER2 expression [23–25]. In addition, celastrol, 11,11′-dideoxy-verticillin (ZH-4B) (*Shiraia bambusicola*), and houttuyninum have been found to suppress* ERBB2 *gene expression in animal models [26–28]. In contrast, our previous studies have demonstrated that Si-Wu-Tang and its constituent (ferulic acid) are able to upregulate HER2 signaling [17, 20].

Estrogen receptor (ER), which is encoded by the* ESR *gene, is a ligand-activated transcription factor essential for sexual development, reproductive functioning, and bone formation. Since the status of ER in breast cancer cells plays an important prognostic role, therapies that antagonize the ER-related signaling, such as tamoxifen, remain important adjuvant treatments after breast cancer surgery [6–8]. Recently, several organisms, such as* Ganoderma lucidum* and* Scutellaria baicalensis*, have been reported to suppress ER expression* in vitro *[29, 30]; while* Salvia miltiorrhiza* was found to activate AKT and inhibit apoptosis in cardiomyoblasts via the estrogen receptor [31]. It is well known that hormone therapies may cause adverse effects on patients, such as insomnia, hot flushes, cancer-related fatigue, and joint pain among breast cancer patients. For this reason, the use of complementary and alternative medicine (CAM) has become popular among patients receiving hormonal therapy [32, 33]. For examples, estrogenic botanical supplements and Chinese herbal remedies are commonly used by breast cancer survivors to relieve their discomfort during hormonal therapy [16, 18, 34]. Recently, herb-drug interaction has become an important issue when treating receptor (+) breast cancer patients.

With regard to functional validation of the present results, our previous* in vitro* studies [17] have shown that Si-Wu-Tang (SWT) not only is able to upregulate the HER2 and ER*α* expression in MCF-7 cells but also stimulates the cell proliferation in BT474 (ER+, HER-2 high), MDAMB231 (ER-, HER-2 low), and SKBR3 (ER-, HER-2 high) mammary duct cell lines. Besides, SWT reversed tamoxifen-induced antiproliferative effects in MCF-7 cells, both* in vitro* and* in vivo*; and SWT also reversed trastuzumab-induced antiproliferative activity in different properties of breast cancer HER2+ cell lines (SK-BR-3 and BT-474) through increased phosphorylation of the cell cycle regulatory protein p27(Kip1) and possibly of the antiapoptosis protein P38 [17, 19].

Jia-Wei-Xiao-Yao-San (JWXYS), which is composed of ten herbs, has been used by Chinese people for centuries and ranks as the first most common Chinese medicine decoction coprescribed with tamoxifen when breast cancer is treated by hormonal therapy [18, 35]. It is of note that some of the herbs in JWXYS are able to upregulate* ERBB2* and* ESR1* gene expression, while JWXYS alone does not induce any significant change in the expression of the above-mentioned genes. We attribute this to the fact that there are drug-drug and drug-cell interactions between these components in JWXYS's composition. Similar but distinct results in terms of synergism were found for VGH-S4, a compound recipe composed of* Curcuma phaeocaulis, Taraxacum mongolicum, Millettia dielsiana,* and* Mentha piperita. *Our results showed that, among the four components, only* Taraxacum mongolicum *was able to significantly upregulate* ERBB2* and* ESR1* gene expression; nevertheless, VGH-S4 had a more potent* ERBB2* and* ESR1* induction effect than* Taraxacum mongolicum* alone, suggesting a positive synergistic effect on* ERBB2* and* ESR1* expression between the herbs in VGH-S4.

In summary, the results obtained from this study provide important information to both Western medical practitioners and TCM doctors who are treating breast cancer using hormonal or targeted therapy.

## Figures and Tables

**Table 1:** Commonly used Chinese single herbs and compound recipes in this study.

Nature	Scientific name	Chinese name	Code
Qi-supplying	*Astragalus membranaceus *	huang qi	A
	*Atractylodes macrocephala *	bai zhu	B
	*Poria cocos *	fu ling	C
	*Glycyrrhiza uralensis *	gan cao	D
	*Agastache rugosa *	huo xiang	E
	*Codonopsis pilosula *	dang shen	F
	*Zingiber officinale *	sheng jiang	G

Blood-regulating	*Angelica sinensis *	dang gui	H
	*Ligusticum chuanxiong *	chuan xiong	I
	*Ziziphus jujuba *	suan tsao jen	J
	*Millettia dielsiana *	ji xue teng	K
	*Curcuma phaeocaulis *	e zhu	L

Heat-clearing	*Folium nelumbinis *	he ye	M
	*Bupleurum chinense *	chai hu	N
	*Mentha piperita *	bo he	O
	*Gardenia jasminoides *	zhi zi	P
	*Paeonia suffruticosa *	mu dan pi	Q
	*Taraxacum mongolicum *	pu gong ying	R
	*Anemarrhena asphodeloides *	zhi mu	S

Yin-nourishing	*Paeonia lactiflora *	bai shao	T
	*Rehmannia glutinosa *	di huang	U
	*Ligustrum lucidum *	nv zhen zi	V

Compound recipes and abbreviations	Si-Wu-Tang	SWT	
	Jia-Wei-Xiao-Yao-San	JWXYS	
	Suan-Zsao-Ren-Tang	SZRT	
	Reduced formula of Suan-Zsao-Ren-Tang	r-SZRT*	
	K'uan-Hsin-Yin	KHY	
	VGH-S4	VGH-S4	

Scientific name is coded by international binomial nomenclature. VGH-S4 is a compound recipe commonly used in Taipei Veterans General Hospital.

*Reduced formula of Suan-Zsao-Ren-Tang (r-SZRT) is composed of those materials in Suan-Zsao-Ren-Tang except for *Ligusticum chuanxiong* which is absent.

**Table 2:** Effects of commonly used Chinese herbs on cytotoxicity.

	Scientific name	Dose range	Cell survival ≦ 80%
Qi-supplying	*Astragalus membranaceus *	0.1~10 *μ*g/mL	(—)
	*Atractylodes macrocephala *	0.1~10 *μ*g/mL	(—)
	*Poria cocos *	0.1~10 *μ*g/mL	(—)
	*Glycyrrhiza uralensis *	0.1~10 *μ*g/mL	(—)
	*Agastache rugosa *	0.1~10 *μ*g/mL	(—)
	*Codonopsis pilosula *	0.1~10 *μ*g/mL	(—)
	*Zingiber officinale *	0.1~10 *μ*g/mL	(—)

Blood-regulating	*Angelica sinensis *	0.1~10 *μ*g/mL	3 *μ*g/mL*
	*Ligusticum chuanxiong *	0.1~10 *μ*g/mL	(—)
	*Ziziphus jujuba *	0.1~10 *μ*g/mL	(—)
	*Millettia dielsiana *	0.1~10 *μ*g/mL	(—)
	*Curcuma phaeocaulis *	0.1~10 *μ*g/mL	(—)

Heat-clearing	*Folium nelumbinis *	0.1~10 *μ*g/mL	(—)
	*Bupleurum chinense *	0.1~10 *μ*g/mL	(—)
	*Mentha piperita *	0.1~10 *μ*g/mL	(—)
	*Gardenia jasminoides *	0.1~10 *μ*g/mL	(—)
	*Paeonia suffruticosa *	0.1~10 *μ*g/mL	(—)
	*Taraxacum mongolicum *	0.1~10 *μ*g/mL	(—)
	*Anemarrhena asphodeloides *	0.1~10 *μ*g/mL	(—)

Yin-nourishing	*Paeonia lactiflora *	0.1~10 *μ*g/mL	10 *μ*g/mL*
	*Rehmannia glutinosa *	0.1~10 *μ*g/mL	(—)
	*Ligustrum lucidum *	0.1~10 *μ*g/mL	(—)

Compound recipes and abbreviations	Si-Wu-Tang	0.1~10 *μ*g/mL	(—)
	Jia-Wei-Xiao-Yao-San	0.1~10 *μ*g/mL	(—)
	Suan-Zsao-Ren-Tang	0.1~10 *μ*g/mL	(—)
	Reduced formula of Suan-Zsao-Ren-Tang	0.1~10 *μ*g/mL	(—)
	K'uan-Hsin-Yin	0.1~10 *μ*g/mL	(—)
	VGH-S4	0.1~10 *μ*g/mL	(—)

Cell cytotoxicity was determined by MTT assay as described in the Methods section. VGH-S4 is a compound recipe commonly used in Taipei Veterans General Hospital.

*P < 0.05 versus vehicle group (*n* ≥ 4~6).

**Table 3:** Effects of Qi-supplying and Blood-regulating herbs on *ERBB2* and *ESR1* gene expression.

Nature	Scientific names	Code	Dose range (*μ*g/mL)	*ERBB2* (fold of control)			*ESR1* (fold of control)		
				≦1.5	1.5–2	≧2	≦1.5	1.5–2	≧2
Qi-supplying	*Astragalus membranaceus *	A	0.3, 1, 3						
	*Atractylodes macrocephala *	B	1, 3, 10		1*				
	*Poria cocos *	C	0.3, 1, 3		0.3	1, 3			
	*Glycyrrhiza uralensis *	D	0.1, 0.3, 1					0.1	0.3, 1
	*Agastache rugosa *	E	0.3, 1, 3		0.3, 1, 3				
	*Codonopsis pilosula *	F	1, 3, 10						3, 10
	*Zingiber officinale *	G	0.1, 0.3, 1						0.1, 0.3, 1

Blood-regulating	*Angelica sinensis *	H	0.1, 0.3, 1		1				
	*Ligusticum chuanxiong *	I	0.1, 0.3, 1			1			0.1, 0.3, 1
	*Ziziphus jujuba *	J	0.3, 1, 3						
	*Mucuna birdwoodiana *	K	0.3, 1, 3						
	*Curcuma phaeocaulis *	L	0.3, 1, 3		3			1, 3	

*ERBB2* and* ESR1* gene expression was determined by luciferase reporter assay as described in the Methods section.

*Number indicates the dose that has effect with statistically significant difference (*P* < 0.05) versus vehicle group (*n* ≥ 6).

**Table 4:** Effects of Heat-clearing and Yin-nourishing herbs on *ERBB2* and *ESR1* gene expression.

Nature	Scientific Names	Code	Dose range (*μ*g/mL)	*ERBB2* (fold of control)			*ESR1* (fold of control)		
				≦1.5	1.5–2	≧2	≦1.5	1.5–2	≧2
Heat-clearing	*Folium nelumbinis *	M	0.3, 1, 3						
	*Bupleurum chinense *	N	1, 3, 10						
	*Mentha piperita *	O	1, 3, 10			3*, 10			
	*Gardenia jasminoides *	P	0.3, 1, 3						1
	*Paeonia suffruticosa *	Q	0.1, 0.3, 1		1				
	*Anemarrhena asphodeloides *	R	0.03, 0.1, 0.3						
	*Taraxacum mongolicum *	S	0.3, 1, 3						

Yin-nourishing	*Paeonia lactiflora *	T	0.3, 1, 3						
	*Rehmannia glutinosa *	U	0.1, 0.3, 1						
	*Ligustrum lucidum *	V	0.3, 1, 3		0.3, 1, 3				

*ERBB2* and *ESR1* gene expression was determined by luciferase reporter assay as described in the Methods section.

*Number indicates the dose that has effect with statistically significant difference (*P* < 0.05) versus vehicle group (*n* ≥ 6).

**Table 5:** Effects of Chinese compound recipes on *ERBB2* and *ESR1* gene expression.

Names	Abbreviation	Dose range (*μ*g/mL)	ERBB2 (fold of control)			ESR1 (fold of control)		
			≦1.5	1.5–2	≧2	≦1.5	1.5–2	≧2
Si-Wu-Tang	SWT	1, 3, 10		10*				1, 3, 10
Jia-Wei-Xiao-Yao-San	JWXYS	1, 3, 10						
Suan-Zsao-Ren-Tang	SZRT	0.1, 0.3, 1			0.1, 0.3, 1		0.1, 0.3, 1	
Reduced formula of Suan-Zsao-Ren-Tang^#^	r-SZRT	0.1, 0.3, 1				0.3	0.1, 1	
Kuan-Shin-Yin	KHY	0.3, 1, 3						0.3, 1, 3
VGH-S4	VGH-S4	0.1, 0.3, 1			0.3, 1			0.1, 0.3, 1

*ERBB2* and *ESR1* gene expression was determined by luciferase reporter assay as described in the Methods section. VGH-S4 is a compound recipe commonly used in Taipei Veterans General Hospital.

^#^Reduced formula of Suan-Zsao-Ren-Tang (r-SZRT) is composed of those materials in Suan-Zsao-Ren-Tang except for *Ligusticum chuanxiong* which is absent.

*Number indicates the dose that has effect with statistically significant difference (*P* < 0.05) versus vehicle group (*n* ≥ 6).
